# Advanced glycation end products of bovine serum albumin-induced endothelial-to-mesenchymal transition in cultured human and monkey endothelial cells via protein kinase B signaling cascades

**Published:** 2010-12-09

**Authors:** Jianli Ma, Ting Liu, Xiaoguang Dong

**Affiliations:** 1 Weifang Medical University, Weifang, China; 2 State Key Lab Cultivation Base, Shandong Provincial Key Lab of Ophthalmology, Shandong Eye Institute, Qingdao, China

## Abstract

**Purpose:**

Advanced glycation end products of BSA (AGE-BSA) participate in the pathogenesis of diabetic vascular disease. However, the role of AGE-BSA in diabetic retinopathy, especially in retinal neovascularization, remains incomplete. This study aimed to determine the contributions of AGE-BSA in the endothelial-to-mesenchymal transition (EnMT) of cultured human and monkey endothelial cell lines and the mechanism that may be related with the transition.

**Methods:**

Monkey choroid-retinal endothelial cells (RF/6A) and human umbilical vein endothelial cells (HUVEC) were cultured in Dulbecco’s modified Eagle’s Medium (DMEM) and Ham’s F12 medium containing 200 mg/l AGE-BSA. The expression of VE-cadherin, β-catenin, vimentin, N-cadherin, and protein kinase B (AKT2) was observed by immunocytochemistry and flow cytometry. Cell motility was determined by migration assays; the endothelial function of the formatting tube was measured by tube formation assays, while the change in the polarity was measured using resistance instruments.

**Results:**

The characteristics of EnMT included loss of endothelial markers of VE-cadherin and β-catenin, which were replaced by mesenchymal markers of vimentin and N-cadherin, enhanced migration and tube formation, and diminished polarity. AGE-BSA contributed to upregulation of the protein expression of VE-cadherin and β-catenin and downregulation of protein expression of vimentin and N-cadherin, leading to enhanced migration and tube formation and diminished polarity. During this process, expression of AKT2 was upregulated.

**Conclusions:**

AGE-BSA can induce EnMT of cultured human and monkey endothelial cells. The signal pathway involving AKT2 may play a role in this process.

## Introduction

In diabetic patients, reducing sugars, including glucose, fructose, and aldotriose, can react nonenzymatically with the amino groups of proteins to form reversible Schiff bases and then Amadori products. These early glycation products become irreversibly cross-linked heterogeneous fluorescent derivatives termed “advanced glycation endproduct” (AGE) [1] after further complex reactions, such as rearrangement, dehydration, and condensation. The accumulation of AGEs in vivo has been found to increase with age and at an accelerated rate in diabetic patients [2]. AGEs have been strongly implicated in the initiation and acceleration of multiple-organ damage in pathological conditions of diabetic etiology, especially the pathogenesis of diabetic microvascular and macrovascular complications [3,4], and non-diabetic etiology, such as cardiovascular [5,6] and renal pathology of aging [5,7]. Tubular cells treated with AGE have myofibroblastic phenotype changes, including elongation, hypertrophy, and separation from neighboring cells [8]. Additionally, tube formation and migration of vascular endothelial cells are dose-dependently stimulated by AGE [9], which provide evidence that AGE can elicit angiogenesis and thereby play an active part in the development and progression of diabetic microangiopathy [10]. This may account for the disabilities and high mortality rates in patients with this disease [11].

Epithelial-to-mesenchymal transition (EMT) was first described in the three-dimensional culture of corneal epithelial cells in the laboratory of Hay in 1982 [12]. Since then many attempts have been made to define this phenomenon. These studies have shown that several types of cells, including retinal pigment epithelial cells, glial cells, fibroblasts, and cells with myofibroblast transformation, have phenotypic changes and thus no longer resemble the normal cell populations from which they originated [13]. This transdifferentiation is a hallmark of EMT, by which epithelial cells lose their epithelial phenotypes and acquire mesenchymal, fibroblast-like properties, show reduced intercellular adhesion, and show increased motility [1,12,14–17]. Recently, some studies have found that, as a special part of epithelial cells, endothelial cells also can transdifferentiate into mesenchymal cells in a similar way which is called endothelial-to-mesenchymal transition (EnMT).

Based on the above, we can suppose that endothelial cells undergo EnMT during angiogenesis in diabetes. Since AGE has important effects on the biologic properties of endothelial cells, especially in subjects with diabetes, is this molecule, at least in part, responsible for the EnMT of vascular endothelial cells in diabetic complications? To address this question, we investigated the effects of the interaction of monkey choroid-retinal endothelial cells (RF/6A) and human umbilical vein endothelial cells (HUVEC) with AGE-modified BSA (AGE-BSA) as a prototype of this class of nonenzymically glycosylated proteins. The mechanism was also detected by measuring the changes of Akt2, which plays a role in human cancer, high-glucose-induced EMT [18], and leptin-modulated EMT [19].

## Methods

BSA and AGE-BSA were purchased from BioVision (Mountain View, CA). Antihuman β-catenin-fluorescein monoclonal antibody, antihuman Akt2-phycoerythrin monoclonal antibody, and antihuman N-cadherin-fluorescein monoclonal antibody were from R&D Systems (Minneapolis, MN). Antihuman vimentin-fluorescein monoclonal antibody was from Santa Cruz Biotechnology (Santa Cruz, CA). Antihuman VE-cadherin-phycoerythrin monoclonal antibody was from eBioscience (San Diego, CA). Fluorescein-conjugated AffiniPure goat antimouse immunoglobulin (IgG) was from ZSGB-BIO (Beijing, China), and millicell cell culture inserts were from Millipore (Boston, MA). Matrigel was from BD Biosciences (San Jose, CA). The monkey choroid-retinal endothelial cell line RF/6A was obtained from the cell bank of the Chinese Academy of Science (Shanghai, China). The HUVEC cell line was from American Type Culture Collection (ATCC number: CRL:1730; Rockville, MD).

### Cell culture

The RF/6A and HUVEC cell lines was routinely cultured in a 1:1 mixture of Dulbecco’s modified Eagle’s medium (DMEM; Invitrogen, Carlsbad, CA) and Ham’s F12 medium (DF12; Invitrogen) supplemented with 10% (v/v) fetal bovine serum (FBS; Invitrogen), 100 U/ml penicillin (Invitrogen), and 100 µg/ml streptomycin (Invitrogen). Cells were maintained at 37 °C, 5% CO_2_ in an incubation cabinet. The culture medium was replaced twice weekly. The fetal calf serum content was lowered to 2% (v/v), and the glucose content was upgraded to 25 mmol/l when the cells were exposed to AGE-modified or unmodified BSA [20]. Cells were grown to confluence in 6-, 24-, or 48-well plates and used within 24 h after confluence was achieved. Cells were separated for subculture with trypsin (0.25%)/EDTA (EDTA; Solarbio Science and Technology Corporation, Beijing, China; 0.02%).

### Flow cytometry

RF/6A and HUVEC cells were seeded onto a six-well plate. When 90% confluence was achieved, cells were treated with AGE-BSA (200 mg/l) or BSA (200 mg/l) for 24 h. Briefly, cells were separated with trypsin/EDTA and washed twice in PBS at 200× g for 5 min before being resuspended and fixed in 0.5 ml cold 4% paraformaldehyde fixative and incubated at room temperature for 10 min. After fixation the cells were washed twice in an isotonic phosphate buffer solution (PBS, 154 mM NaCl, 16.8 mM Na_2_HPO_4_, 2.6 mM NaH_2_PO_4_,pH 7.4) supplemented with 0.5% BSA by centrifuging at 200× g for 5 min, resuspended in 900 µl of 0.3% Triton X-100, incubated for 20 min at 4 °C, centrifuged at 200× g for 5 min, and washed twice in PBS/BSA buffer. After the second wash, approximately 200 µl of buffer was left in the tube and cells were resuspended. The conjugated antibodies of VE-cadherin, β-catenin, N-cadherin, Akt2, and vimentin were added to the suspension before the cells were incubated for 45 min at room temperature in the dark. After washing twice using PBS/BSA buffer, the cells were resuspended in each tube with 200 µl of PBS for the final flow cytometric analysis.

### Immunocytochemistry

RF/6A and HUVEC cells were seeded onto a 48-well plate. When confluence was achieved, the cells were treated with AGE-BSA (200 mg/l) or BSA (200 mg/l) for 24 h. Briefly, cells were rinsed in PBS twice and fixed in 0.5 ml cold 4% paraformaldehyde for 20 min. After rinsing in PBS three times, 5 min per time, the cells were permeabilized with 0.3% Triton X-100 for 20 min and then rinsed three times in PBS, 5 min per time. The plate was incubated with primary antibody to VE-cadherin, β-catenin, N-cadherin, Akt2, or vimentin overnight at 4 °C before being rinsed and incubated with fluorescein-conjugated AffiniPure goat antimouse IgG for 1 h at room temperature. Stained cells were visualized, and images were captured using a laser confocal microscope (Nikon/C1 Plus; Nikon, Tokyo, Japan).

### Migration assays

RF/6A and HUVEC cells were seeded onto a six-well plate, grown to confluent monolayers, and treated with AGE-BSA (200 mg/l) or BSA (200 mg/l) for 24 h. A scratch wound was then inflicted on the monolayer with a p20 pipette tip. The ability of the RF/6A and HUVEC cells to close the wound space was used to assess the migratory ability of the cells at 24 and 48 h after the scratch. Light microscopic images were taken when the scratch was made and at 24 h.

### Transendothelial electrical resistance

RF/6A cells were seeded onto millicell cell culture inserts (12 mm in diameter, 0.4 µm poly-carbonate-filter [PCF]) and mounted on a 24-well plate for 10 days before treatment. Medium containing AGE-BSA (200 mg/l) or BSA (200 mg/l) was added to the upper (400 µl) and lower chambers (600 µl) of the millicell cell culture inserts, while DF12 medium without drugs was added to the control group. The cells were incubated at 37 °C, 5% CO_2_ for 48 h, and the medium was replaced every day. Transendothelial electrical resistance (TER) of the monolayers was measured using a resistance instrument (Cat.No. MERS 00001, 0–1999Ω ; Millipore) immediately, at 24 h, and at 48 h after AGE-BSA or after BSA was mixed into the medium. A group without cells was set as blank controls [21]. Each group was measured three times at each time point, and the average was used to calculate the TER by the formula TER (Ω·cm2)=(the average of every group–the average of the blank control group)×0.6 (the undersurface area of the millicell cell culture inserts).

### Tube formation assays

HUVEC cells were seeded on matrigel-coated 96-well plates. The cells were treated with medium without drugs, medium containing AGE-BSA (200 mg/l), or medium containing BSA (200 mg/l), and then the cells were incubated at 37 °C, 5% CO_2_ for 6 h. Tube formation was quantified by counting the number of connected cells in randomly selected fields at 100× magnification.

### Statistical analysis

One-way ANOVA (ANOVA) was used for comparison between the AGE-BSA group and the control groups. Paired-sample *t* test was used for the comparison of cell polarity before and after treatment with different media. A p<0.05 was considered significantly different.

## Results

### Loss of endothelial markers and acquisition of mesenchymal markers

**Figure 1:**
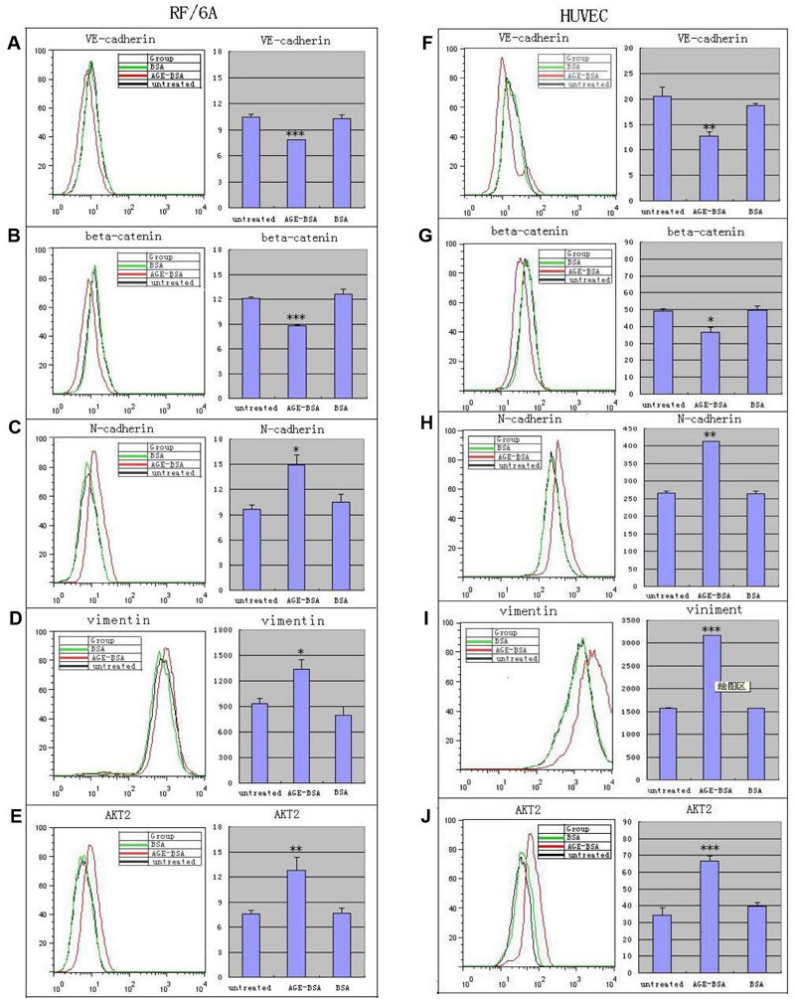
Flow cytometry was used to illustrate altered expression of VE-cadherin, β-catenin, vimentin, N-cadherin, and protein kinase B (AKT2) in monkey choroid-retinal endothelial cells (RF/6A) and human umbilical vein endothelial cells (HUVEC) after the treatment with advanced glycation end products of BSA (AGE-BSA; 200 mg/l) for 24 h. For RF/6A cells, there were decreased expression of markers of endothelial cells, VE-cadherin (**A**) and β-catenin (**B**), and increased expression of markers of mesenchymal cells, N-cadherin (**C**) and vimentin (**D**). Similar to the RF/6A cells, the protein expression of VE-cadherin (**F**) and β-catenin (**G**) in the HUVEC cells was downregulated, and the expression of N-cadherin (**H**) and vimentin (**I**) in the HUVEC cells was upregulated. At the same time, the expression of Akt2 in the both cells increased (**E**, **J**). *p<0.05, **p<0.01, ***p<0.001.

**Figure 2:**
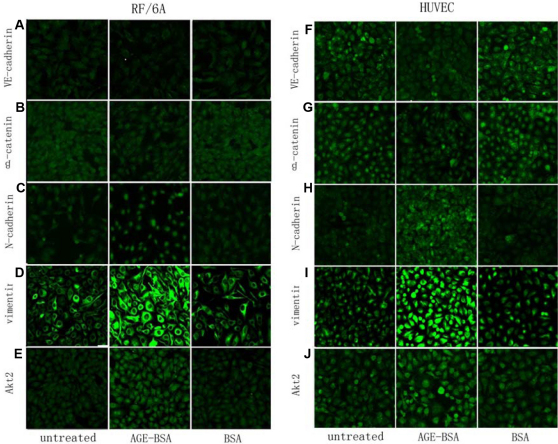
Immunocytochemistry was used to illustrate altered expression of VE-cadherin, β-catenin, vimentin, N-cadherin and protein kinase B (AKT2) in monkey choroid-retinal endothelial cells (RF/6A) and human umbilical vein endothelial cells (HUVEC) after the treatment by advanced glycation end products of BSA (AGE-BSA; 200 mg/l) for 24 h. For RF/6A cells, there were decreased expression of markers of endothelial cells, VE-cadherin (**A**) and β-catenin (**B**), and increased expression of markers of mesenchymal cells, N-cadherin (**C**) and vimentin (**D**). Similar to the RF/6A cells, the protein expression of VE-cadherin (**F**) and β-catenin (**G**) in the HUVEC cells was downregulated, and the expression of N-cadherin (**H**) and vimentin (**I**) in the HUVEC cells was upregulated. At the same time, the expression of Akt2 in the both cells increased (**E**, **J**). Additionally, in the RF/6A cells, the decreased part of β-catenin was mainly in the cytoplasm, while the protein expressed in the nucleus changed little (**B**); after treatment with AGE-BSA, N-cadherin expression was upregulated in the nucleus and downregulated in the intracytoplasm (**D**). In the HUVEC cells, there were no such phenomena.

In RF/6A cells, upregulation of the protein for N-cadherin and vimentin (Figure 1C,D and Figure 2C,D) were noted by immunocytochemistry and flow cytometry after 24-h treatment. Moreover, by immunocytochemistry, N-cadherin protein in the nucleus was significantly increased but that in the cytoplasm decreased and even disappeared (Figure 2C). The expression levels of VE-cadherin and β-catenin in the cytoplasm decreased after 24-h treatment (Figure 1A,B and Figure 2A,B), and the β-catenin level in the nucleus did not show much change (Figure 2B). The total expression of VE-cadherin was low not only in the treated group but also in the control groups; total expression of VE-cadherin in the treated group was lower (Figure 2A).

Similar to RF/6A cells, after treatment with AGE-BSA, upregulation of the protein for N-cadherin and vimentin (Figure 1H,I and Figure 2H,I) in HUVEC cells were noted by immunocytochemistry and flow cytometry, while the expression levels of VE-cadherin and β-catenin decreased significantly (Figure 1F,G and Figure 2F,G). In contrast with the RF/6A cells, the change in the expression level of proteins was not different in the cytoplasm and the nucleus.

### Enhanced migration after treatment

**Figure 3:**
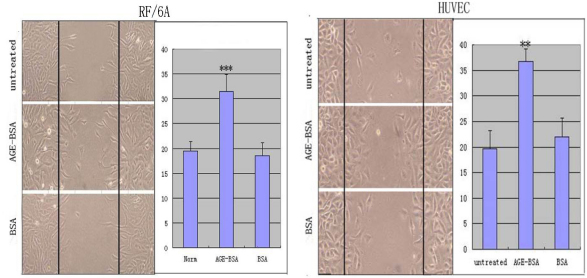
Advanced glycation end products of BSA (AGE-BSA) induced enhanced migration in transdifferentiated monkey choroid-retinal endothelial cells (RF/6A) and human umbilical vein endothelial cells (HUVEC). Light microscopic images shows the increased migratory ability of cells after 24 h of treatment with AGE-BSA. The images were taken at 24 h after the scratch was applied. The migratory ability of the transdifferentiated cells was most obvious in the cells treated with AGE-BSA after scratch injury. ***p<0.001.

After treatment with a different medium for 24 h, there were more cells in the scratch inflicted on the monolayer in the AGE-BSA group compared with the two control groups. The migration ability of the RF/6A and HUVEC cells was enhanced significantly by AGE-BSA (Figure 3).

### Decreased polarity of monkey choroid-retinal endothelial cells

On the day 10 of the culture, there was no significant difference in cell polarity between the AGE-BSA group and the two control groups (p=0.961 and 0.873, respectively) and between the two control groups (p=0.866). Compared with the two control groups, the polarity of the AGE-BSA group decreased significantly at 24 h (p=0.027 and 0.031, respectively) and 48 h (p=0.015 and 0.012, respectively) after treatment with AGE-BSA. The difference in polarity between the two control groups was not significant at the two time points (p=0.715 and 0.969, respectively).

The polarity of cells decreased significantly at 24 h and 48 h after treatment with AGE-BSA compared with that before treatment (p=0.002 and 0.001, respectively), and the difference between 24 h and 48 h was significant (p=0.011). There was no difference in polarity between the two control groups (Table 1).

**Table 1:** The TER of the three group at different time after the treatment (Ω·cm2)

**Groups**	**n**	**Before treatment**	**24 h after treatment**	**48 h after treatment**
Norm	4	37.84±3.79	36.79±3.00	36.68±4.18
AGE-BSA	4	37.95±2.21	31.31±2.36**	28.54±2.45**
BSA	4	38.29±3.40	37.73±3.91	36.56±3.75

Paired-sample *t*-test was used for comparison of cell polarity before and after treatment with different media at different times, which revealed a significant decrease of cell polarity after 24-h and 48-h AGE-BSA treatment, but there was no significant difference between the normal and BSA groups. **p<0.01.

### Enhanced tube formation of human umbilical vein endothelial cells

**Figure 4:**
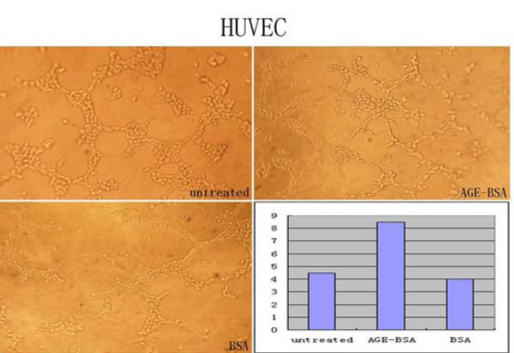
The tube formation ability of the human umbilical vein endothelial cells (HUVEC) was enhanced significantly by advanced glycation end products of BSA (AGE-BSA). After treatment with different media for 6 h, the number of the tube formatted by connected cells in the AGE-BSA group was larger than that in the two control groups. The tube formation ability was enhanced evidently after AGE-BSA treatment.

After treatment with different medium for 6 h, the number of tubes formatted by connected cells was more in the AGE-BSA group than that in the two control groups. Tube formation ability of the HUVEC cells was enhanced significantly by AGE-BSA (Figure 4).

### Increased protein kinase B expression after treatment

There was an obvious upregulation of the protein level of Akt2 in RF/6A cells after treatment with AGE-BSA for 24 h. This result was verified by flow cytometry, which showed a significant difference between the AGE-BSA group and the control groups, while there was no significant difference between the two control groups (p=0.749; Figure 1E and Figure 2E). The same result was found in HUVEC cells after treatment with AGE-BSA for 24 h (Figure 1J and Figure 2J).

## Discussion

Microvessels are composed of two types of cells, endothelial cells and pericytes, and have been known to show both functional and structural abnormalities during prolonged diabetic exposure, resulting in deleterious effects on the organs that they supply [22–24]. It was reported that AGE exerts a growth inhibitory effect and a cell type-specific immediate toxicity on pericytes through interactions with their receptor for AGE and leads to pericyte loss. The AGE-induced decrease in pericyte number would indirectly cause angiogenesis [25,26].

AGEs in circulation and on the subendothelium interact directly with endothelial cells through a specific binding system. The functional consequences of AGE-endothelial interactions include decreasing the expression of VE-cadherin [27] and increasing the expression of the vimentin, α-smooth muscle actin, and matrix proteins [8], leading to the disruption of the vascular endothelial cadherin complex of the endothelial cells and increasing vascular permeability [6].

Some investigations have demonstrated that sustained hyperglycation results in a gradual AGE accumulation within retinal cellular and extracellular structures, including basement membrane, endothelium, pericytes, and smooth muscle cells of diabetic rats. AGE epitopes occur first in the retinal vascular basement membrane of newly diabetic rats. AGE formation has been suggested as a plausible factor for the irreversible components of diabetic complication [23,28]. In this study, we examined the effects of AGE-BSA on RF/6A and the HUVEC cell line and showed a loss of endothelial markers and a gain of mesenchymal markers. At the same time, cells had increased motility, enhanced tube formation, and decreased polarity. EnMT of endothelial cells is associated with decreased expression of endothelial cell markers, which is in conjunction with increased expression of mesenchymal cell markers, N-cadherin, and vimentin [29–33]. Endothelial cells occupy a central anatomic position in the microvessels. Therefore, AGE-induced endothelial cell changes may contribute in part to diabetic neovascularization, which is an important cause leading to diabetic complication.

VE-cadherin, as an integral membrane glycoprotein expressed exclusively in endothelial cells [34–36], clusters at endothelial cell junctions, mediates cell adhesion in a calcium-dependent manner, inhibits cell proliferation, and decreases cell permeability and migration when overexpressed in various cell types [37–39]. In addition, VE-cadherin functions as a plasma membrane attachment site for the cytoskeleton through its interactions with the cytoplasmic protein β-catenin [40], which is an armadillo family member that binds intracellularly to the VE-cadherin cytoplasmic domain and also a structural protein involved in cell–cell adhesion [41]. The VE-cadherin–β-catenin complex is essential in endothelial cells for normal vascular patterning [42,43]. It is a target of permeability increasing agents. The dissociation of this complex leads to the reduction of cell adhesion and increase of cell permeability [44]. Previous studies have shown that the catenin–cadherin complex is required to maintain the mammary gland architecture and influences polarity, cell fate, and motility of epithelial cells [45]. Perturbation of the β-catenin–E-cadherin complex can result in the nuclear localization of β-catenin, which is associated with increased vimentin and vascular endothelial growth factor expression along with a potentially more invasive phenotype [46]. This phenomenon was also observed in RF/6A but not HUVEC cells. In RF/6A cells, the decreased part of β-catenins was almost in the cytoplasm, while the expression in the nucleus changed invisibly, which is similar to the findings of Mironchik et al. [32]. On the other hand, the formation of AGEs has been strongly implicated in the endothelial dysfunction associated with microvascular and macrovascular complications that accompany diabetes and normal aging by altering the structural of endothelial junction organization [4,20]. In the current study, exposure of RF/6A and HUVEC cells to AGE-BSA for 24 h induced a decrease in the amount of VE-cadherin and the major component of the VE-cadherin complex that were linked directly to the cytoplasmic tail of VE-cadherin and β-catenins. This, in turn, could induce perturbations to properties of the endothelium and thereby contribute to vascular dysfunction. This finding is in agreement with the idea that AGE-BSA greatly alters the organization of the endothelial VE-cadherin complex of HUVEC [20].

One study has found that, after withdrawal of AGE-BSA, the barrier properties caused by AGE-BSA returned to baseline, with a concomitant increase in the VE-cadherin complex content. This suggests that AGEs must be constantly present to exert the observed effects. This is in accordance with AGE physiology, as AGE proteins accumulate in the vasculature with aging and at an accelerated rate in diabetes. The effects exerted by AGEs should be related to their chronic presence in the diabetic milieu [20].

Normal endothelial cells express only a small amount of N-cadherin and vimentin. Vimentin is the major intermediate filament of mesenchymal cells and has viscoelastic properties that allow it to stabilize cell structure in migration. Elevated expression of vimentin contributes to the biologic properties of cells, including enhanced proliferation and motility [47], which are the biologic properties of mesenchymal cells. Decreases in the levels of cellular cadherins may affect other cadherins presenting in endothelial cells, namely neural (N)-cadherin. This is a classical cadherin presenting in significant amounts in endothelial cells [48]. It interacts with catenins and the actin cytoskeleton to promote homotypic endothelial cell–cell adhesion. In endothelial cells, N-cadherin is not located at cell–cell contacts but remains diffuse on the cell membrane. VE-cadherin appears to play a predominant role over N-cadherin in promoting homotypic endothelial cell–cell adhesion and barrier properties, while N-cadherin may be involved in the interaction of endothelial cells with other cell types in the vasculature [49]. In our study, after exposure to AGE-BSA for 24 h, N-cadherin expression was evidently upregulated in both cell lines. It is interesting that the increased part of N-cadherin in RF/6A cells was limited in the nucleus, while the expression in the cytoplasm decreased and even disappeared, which was not found in HUVEC cells. In epithelial malignancies, concurrent with the loss of E-cadherin expression, gain of N-cadherin expression has been shown to be important in the regulation of cell migration, invasion, and survival [50–52]. In addition, aberrant N-cadherin expression in the nucleus is required for cell migration during transforming TGFβ1-stimulated epithelial-to-mesenchymal transformation [53]. In this study, we noticed different changes in the two cell lines, and further investigations are needed to explain this difference.

It should be noted, however, that the cells used in this study were cell lines, not primary endothelial cells. In general, endothelial cell lines may have already acquired mesenchymal characteristics, and the degree of mesenchymal phenotype expressed is different from one cell line to another. It is therefore not justified to expect that expression of the same type of endothelial molecules in various endothelial cell lines or expression of the same mesenchymal molecules in various mesenchymal cell lines will be comparable [54]. In fact, RF/6A is considered to be a typical normal endothelial cell line, which expresses a low level of VE-cadherin but a high level of the mesenchymal markers, N-cadherin and vimentin. In this study, the expression of the endothelial molecules was downregulated, while the mesenchymal molecules were upregulated further after treatment with AGE-BSA. Moreover, our results were obtained at only 24 h after treatment when the contact among cells was perhaps insufficient. It is not surprising that the protein associated with contact had a low expression.

At the cellular level, EnMT includes two distinct steps: decreased intercellular adhesion (to dissociate from the endothelial cellular sheets) and increased cell motility (to migrate into connective tissues). Endothelial cells are characterized by two major histological findings [55]. First, they have the ability to form barriers between the two tissue compartments that the endothelium separates. Second, the plasma membrane of the cell is intrinsically polarized into apical and basolateral domains. Endothelial tightness is maintained by formation of specialized structures known as tight junctions. Apart from restricting the paracellular movement of molecules and ions across the endothelial sheet, tight junctions also have a role in the maintenance of the apical/basolateral polarity [56,57]. By decreasing VE-cadherin in the membrane domain facing the apical compartment and increasing apical accessibility via tight junctions, AGE-BSA decreases the degree of polarization in RF/6A and HUVEC cells and decreases intercellular adhesion. Enhanced migration is the other important characteristic of EnMT. During EMT, cellular transformation drives epithelial remodeling by converting cohesive, stable, epithelial layers into individual, motile, mesenchymal cells, allowing the efficient migration of cohesive epithelium that maintains internal organization [58]. In our study, after treatment with AGE-BSA for 24 h, the migration of RF/6A and HUVEC cells was enhanced, which was associated with the disruption of the VE-cadherin–β-catenin complex and the gain of N-cadherin and vimentin [59]. During this process, cellular transformation resulted in a loss of apical basal polarity, followed by a shift in cytoskeletal dynamics toward the mesenchymal phenotype. This allowed the cells to move freely [18], leading to the dysfunction of the microvessels. From the tube formation study of HUVEC cells, we also found that the decreased polarity and increased motility resulted in the enhanced ability of tube formation, which could facilitate the formation of neovascularization in vivo. This result was consistent with the demonstration that AGE-BSA significantly increased cell migration and tube formation in retinal endothelial cells [60].

The mechanism of EMT has been investigated by many researchers, but EnMT has only had limited investigation. In the present study, we noticed that after treatment with AGE-BSA, the Akt2 expression in RF/6A cells was visibly upregulated. Previous studies revealed that during EMT, engagement of E-cadherin in homophylic calcium-dependent cell–cell interactions results in rapid PI3K-dependent activation of Akt, indicating that E-cadherin can initiate outside-in signal transducing pathways that regulate the activity of PI3K and Akt [17,61]. An activated PI3K/Akt pathway is well documented for various human malignancies, sometimes correlates with an aggressive phenotype [62], and plays a central role in EMT [63–66]. The upregulation of the Akt-involved signaling pathway in this study participated, at least partly, in AGE-BSA-induced EnMT, which may play an important role in the transition. It was reported that the expression of Akt in mesenchymal cells signifies a potential role in maintenance of the mesenchymal phenotype after EMT or participation in invasion-associated cellular activities [67]. In contrast, with activation of the Akt-involved pathway during the EMT, Song et al. provided evidence that N-cadherin acts as a negative regulator of cell proliferation and survival in osteoblasts via interaction with LRP5 and attenuation of Wnt, ERK, and PI3K/Akt signaling pathways [61].

In conclusion, AGE-BSA can induce the endothelial-to-mesenchymal transition of RF/6A and HUVEC cells. The signal pathway involves AKT2, which may play a certain role in this process. Further investigations are required to discuss the inner mechanism of AGE-BSA-induced EnMT.
